# The role of facial distinctiveness in the prioritisation of targets in disjunctive dual-target face search

**DOI:** 10.1186/s41235-024-00589-z

**Published:** 2024-09-13

**Authors:** Emma Smillie, Natalie Mestry, Dan Clark, Neil Harrison, Nick Donnelly

**Affiliations:** 1 Liverpool Hope University, Liverpool, L16 9JD UK; 2 University of Bolton, Bolton, BL3 5AB UK; 3 Bournemouth University, Bournemouth, BH12 5BB UK

**Keywords:** Visual search, Face encoding, Dual-target search, Face memory

## Abstract

Two experiments explored the search for pairs of faces in a disjunctive dual-target face search (DDTFS) task for unfamiliar face targets. The distinctiveness of the target was manipulated such that both faces were typical or distinctive or contained one typical and one distinctive target. Targets were searched for in arrays of eight faces. In Experiment 1, participants completed a DDTFS block with targets learnt over the block of trials. In Experiment 2, the dual-target block was preceded by two training blocks of single-target trials. Participants also completed the upright and inverted long-form Cambridge Face Memory Test (CFMT+). The results showed that searching for two typical faces leads to one target being prioritised at the expense of the other. The ability to search for non-prioritised typical faces was associated with scores on the CFMT+. This association disappeared when faces were learnt before completing DDTFS. We interpret the findings in terms of the impact of typicality on face learning, individual differences in the ability to learn faces, and the involvement of capacity-limited working memory in the search for unfamiliar faces. The findings have implications for security-related situations where agents must search for multiple unfamiliar faces having been shown their images.

## Public Significance Statement

Security officers (e.g. police officers) are often required to be on the lookout for specific individuals or suspects. The present study shows that there is a profound challenge in finding unfamiliar targets when searching for more than one face at the same time. Importantly, the nature of this challenge depends on two factors: first, the relative typicality of the faces that are being sought at the same time, and second, the face processing ability of the searchers. The findings have implications for the design of the job roles and the recruitment of security officers tasked with searching for specific individuals.

## Introduction

In some situations, observers might be required to look out for a specific person of interest. For the general public, the person of interest might be a missing person seen on a poster that they might come across incidentally while going about their daily life (Moore & Lampinen, 2019). For security personnel, the person of interest might be a shoplifter or a terrorist suspect who is being actively sought. Despite being actively sought, a target face may not be especially familiar, beyond being seen in an image, set of images or video. It is this situation, searching for a previously unfamiliar face, that the current study investigates.

The task of actively detecting a specific target in a crowded space is a visual search task. Given that attentional guidance to faces is very limited (Mestry et al., 2017; Towler et al., 2016), it is a form of visual search where each face in the crowd is a potential target until inspected and rejected. Matching an image of an unfamiliar face and a real person is difficult (Bruce et al., 1999; Burton et al., 2010; Davis & Valentine, 2009; Kemp et al., 1997; Megreya & Burton, 2006). It is a task that often leads to identification errors, even for those who conduct this task frequently, such as border force officials (White et al., 2014). The visual search for an unfamiliar target face is, therefore, likely to result in targets being missed. The current studies were motivated by the need to increase our understanding of stimulus and individual-level factors that influence performance on this task.

In some types of surveillance situations, it may be necessary to search for two or more faces at the same time: for instance, a police officer may need to be on the lookout for two or more suspects at an event. However, there are relatively few studies exploring the simultaneous search for more than one target face (e.g. Bindemann et al., 2012; Dunn et al., 2021). In one study that is especially pertinent to the present case, Mestry et al. (2017) had participants to perform a disjunctive dual-target face search (DDTFS) for either of two hitherto unfamiliar face targets (henceforth unfamiliar face targets) and compared the accuracy and speed of search in this condition to single-target baselines. Participants searched for the same two faces across dual-target and single-target conditions such that target faces were repeated over the duration of the study. Mestry reported two critical findings. First, DDTFS was more difficult (i.e. slower and less accurate) than single-target face search. Second, DDTFS was associated with one target being prioritised over the other, especially when faces were highly unfamiliar (i.e. at the beginning of the study). Mestry et al. proposed that the need to prioritise one of the unfamiliar face targets in DDTFS resulted from a limitation in working memory (WM) capacity for faces, with the capacity of WM for faces being limited to 1–2 faces (Mestry et al., 2017; Towler et al., 2016).

The target faces in DDTFS can differ on several different dimensions (e.g. familiarity, ethnicity). Of particular interest in the present study is the dimension of typicality, which refers to the degree to which a face deviates from an average face. Experimental studies have consistently shown that distinctive faces are learnt faster and recalled more accurately than typical faces (Cohen & Carr, 1975; Ellis et al., 1988; Light et al., 1979; Newell et al., 1999). One consequence of distinctive faces being learnt faster than typical faces is that their target templates will more quickly be supported by long-term memory (Cowan, 1988, 1998; Wolfe, 2012). The specific mechanism that produces better memory for distinctive items is debated. Some suggest that distinctive stimuli are prioritised and subsequently receive enhanced processing at encoding (e.g. Schmidt, 1991). Others argue that the more distinctive items are simply easier to differentiate from other less distinctive objects at retrieval (Kelley & Nairne, 2001). Irrespective of the specific mechanism, without the support of long-term memory, the search for unfamiliar typical target faces is likely to rely on WM for longer than that of distinctive faces.

The present study investigates the role of face typicality in DDTFS by manipulating the pairing of typical and distinctive faces as targets to be searched for in arrays of faces. We predicted that the accuracy and speed of DDTFS will be influenced by the typicality of the face targets. Specifically, we predicted increased prioritisation of one face target over the other when searching for pairs of typical faces compared to cases when at least one target is distinctive. This is because of the increased reliance for typical, relative to distinctive faces, on the capacity-limited WM and the need to manage the consequences of this limitation on target search.

There are known to be individual differences in the ability to recognise and identify faces that can be measured by the long-form Cambridge Face Memory Task (CFMT + Russell et al., 2009). Performance on the CFMT + has been shown to be associated with performance on other face-related tasks, including the ability to search for a face in the crowd (Davis et al., 2018; Thielgen et al., 2021). Our interest in the present study is in whether individual differences in the ability to perform DDTFS are related to performance on the CFMT+. Specifically, we investigate whether there is an association between the accuracy and speed of performance in the DDTFS and performance on the CFMT+, and whether this potential association differs for faces prioritised and not prioritised by searchers. We predict that those scoring higher on the CFMT + will have the face processing skills to encode prioritised and non-prioritised faces faster than those scoring lower on the CFMT+. We also predict that individuals scoring highly on the CFMT + will be able to encode faces quickly such that they can reallocate resources to the non-prioritised face target. If so, then scores on the CFMT + may be more strongly associated with the accuracy and speed of search for non-prioritised than prioritised faces. In addition, we hypothesise that association between performance on the DDTFS and the CFMT + is likely to be strongest when search relies most on the capacity-limited WM. Specifically, we predict that the association between search for non-prioritised faces and the CMFT + will be strongest when searching for two typical faces.

An important factor to consider when making predictions about measures that might be associated with the speed and accuracy of the search for prioritised and non-prioritised faces is the nature of the facial representations used in the search. Evidence shows that when searching for unfamiliar faces the non-prioritised faces may be searched for using featural information present in faces (e.g. heavy eyebrows) as opposed to more holistic information representing whole faces (cf. Lobmaier & Mast, 2007). We gained an estimate of the kind of facial features used by participants to search for and identify faces by getting them to complete the CFMT + both upright and inverted. The face inversion effect (Yin, 1969) shows upright faces are processed differently to inverted faces. If the ability to search for non-prioritised faces is more strongly associated with scores on the upright CFMT +, then this would be consistent with the search for non-prioritised faces being conducted using holistic facial information as opposed to simple facial features. In contrast, if the ability to search for non-prioritised faces is more strongly associated with scores on the inverted CFMT +, then this would be consistent with the search for non-prioritised faces being conducted using facial features.

In summary, the current studies investigated stimulus and individual-level factors involved in DDTFS. Participants performed a DDTFS where they searched for two unfamiliar faces in an array of faces. First, we investigated the role of the relative distinctiveness of target faces in the prioritisation of faces. Second, we tested whether individual differences in face recognition ability, as indexed by scores on the CFMT+, were associated with the accuracy and speed of search for prioritised and non-prioritised faces.

## Experiment 1

In Experiment 1, three groups of participants completed a simultaneous DDTFS for faces followed by the upright and inverted versions of the CFMT + (henceforth CFMT + U and CFMT + I). The group searching for two distinctive faces were referred to as the Distinctive group, the group searching for two typical faces were referred to as the Typical group, and the group searching for a typical and a distinctive face were referred to as the Mixed group.

## Method

### Participants

The required sample size was determined via an a priori power analysis, calculated using the main effect of distinctiveness. This was taken from previous work exploring the effect of distinctiveness on the discrimination of unfamiliar faces (Smillie et al., 2022) $\eta_{g}^{2}$ = 0.11. Generalised eta was converted into partial eta ($\eta_{p}^{2}$ = 0.4) for G* Power input and resulted in a large effect size. As the previous work used an easier task, we opted for a conservative approach and recalculated $\eta_{p}^{2}$ = 0.04 ($\eta_{p}^{2}$. = 0.4 divided by 10) to have sufficient power to detect and a small-to-medium effect size of *F* = 0.2. All calculations were performed using G* Power (Faul et al., 2007—version 3.1.9.4) software with an alpha level of 0.05 and a power of 0.95, and this resulted in a minimum sample size of 96 to have sufficient power to detect the main effect of distinctiveness. The study aimed to recruit 108 participants to allow for possible withdrawals or incomplete data sets due to the experiment being conducted online.

In the current study, the primary effect of interest is for the between-participant factor of distinctiveness; however, for completeness, we also calculated the sample size required for the effect of the within-participant factor of Trial and its interaction by distinctiveness. For the main effect of Trial, we estimated the sample size using the effect size of the main effect of target absent vs target present reported in Mestry et al. (2017), $\eta_{g}^{2}$ = 0.074. This was converted to Cohen’s *F* = 0.7 and required a sample size of 12 to achieve power of 0.95 with an alpha of 0.05. For the effect of distinctiveness by Trial interaction, we were not able to find a comparable effect size to use to calculate the required sample size. As such, we estimated that with a small-to-medium effect size *F* = 0.15 and alpha of 0.05, we would require 111 participants to achieve power of 0.80 to detect the interaction and 177 participants to achieve power of 0.95. A sample size calculation was also completed for the correlation analysis to detect a small effect (0.3), with an alpha of 0.05, and we would require 84 participants to achieve power of 0.8 or 138 participants to achieve power of 0.95.

A total of 108 participants were recruited and randomly allocated into one of three groups (*n* = 36 per group). Participants ranged in age between 18 and 51 years (*M* = 21.86, SD = 6.19), and 22 participants were male, one participant self-reported as gender fluid and a second preferred not to say, the remaining participants identified as female. Participants were recruited via the Liverpool Hope and Bournemouth University participation schemes. Students received course credits for their participation. All participants reported normal colour vision and normal or corrected to normal visual acuity. Full ethical approval was granted from Liverpool Hope University and Bournemouth University. All participants consented to their data being published as part of the research project.

### Apparatus and materials

All participants completed upright (CFMT + U) and inverted (CFMT + I) versions of the Cambridge Face Memory Test long form (Russell et al., 2009) and the DDTFS task. The CFMT + (upright and inverted versions) were administered using Qualtrics XM Platform (2020 - www.qualtrics.com).

#### DDTFS task

For the DDTFS task, participants searched for two target faces in an array of faces. They had to respond whether one target was present, or both targets were absent. To create the task, 132 white male faces were selected from the Glasgow Unfamiliar Face Database (GUFD, Burton et al., 2010). As there is some evidence that female participants would have an advantage over male participants when identifying female but not male faces (Lewin & Herlitz, 2002; Megreya et al., 2011), the selection of faces for the stimulus set was limited to white males. There were no constraints on age, hair colour or style, and facial adornments such as piercing or stubble to allow for realistic variations in distinctiveness. Faces were cropped in an oval annulus to remove most facial hair. Cropping faces ensured that the face outline and external features, commonly relied on in the identification of unfamiliar faces (Ellis et al., 1979), were not used as cues. As we wanted participants to familiarise themselves with the target faces, we chose to do this as internal features are utilised more for familiar face recognition, where performance is superior (Ellis et al., 1979). A reason for this is that internal features are more reliable for identifying individuals as they remain similar across instances, whereas external features are more variable (Burton et al., 2005). All faces were presented in full colour against a white background and text instructions appeared in black. Target faces appeared in the array in 50% of trials with each target appearing in half of the target-present trials.

**Fig. 1:**
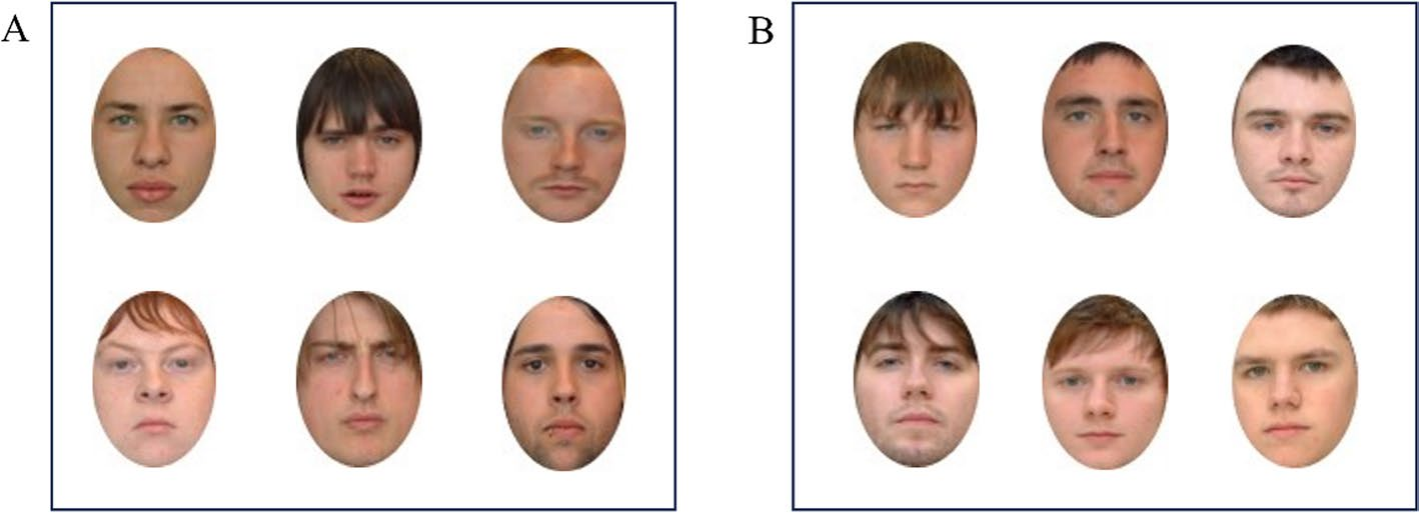
The distinctive and typical faces used as targets in experiment 1 and experiment 2. *Note* An image showing faces rated as most distinctive (panel A) and most typical (panel B). Images sourced from set C1 of the Glasgow Unfamiliar Face Database

The faces were rated for distinctiveness in a pre-study by an independent set of participants (*N* = 24, aged between 18 and 37 years [*M* = 20.58, SD = 4.65]) using a 7-point Likert scale. All participants in the pre-study were White, and 6 were male. The six most distinctive (ranging from 5.13 to 5.95, *M* = 5.54, SD = 0.30) and six most typical faces (ranging from 2.25 to 2.33, *M* = 2.3, SD = 0.04, Fig. 1) were selected as targets (*t*(11) = 7.05, *p* < 0.001, *d* = 2.04).

The remaining 120 faces were used as distractors in the probe arrays with distinctiveness ratings falling between the extremes (ranging from 2.340 to 5.13, *M* = 3.63, SD = 0.62). The distractors appearing in the search array were randomly selected on a trial-by-trial basis. They were selected irrespective of distinctiveness.

The faces used as targets were held constant for each participant but varied systematically across participants with both faces drawn from the pool of distinctive or typical faces or with one face drawn from each pool. No participant searched for the same pair of faces as another. Participants searched for either two distinctive targets, two typical targets, or a typical and a distinctive target. Different images of each target were used in the target preview and test phases. The faces shown as target previews were taken from set C1 of the Glasgow face database and presented adjacent to each other in the target preview. The side on which target faces appeared in the preview was counterbalanced across trials for each participant. Faces used in the search array (both targets and distractors) were taken from set C2 of the Glasgow face database. Using different images for target preview and search arrays ensured that participants could not rely on simple image matching to find a target.

Participants completed 10 practice trials followed by 240 experimental trials of simultaneous DDTFS. Half of the experimental trials were target-present (60 trials for each target), and half were target absent. Search arrays contained eight faces. In target-present trials, there was one target face and seven distractors. On target-absent trials, there were eight distractor faces. Images for the practice trials were sourced from an online image search of Finnish celebrities that would be unfamiliar to participants.

The DDTFS task was built in PsychoPy (Pierce, 2007 Version 2020.2.8) with additional code written in Python language and hosted on Pavlovia (www.pavlovia.org). Target-present and target-absent responses were made using the ‘y’ and ‘n’ keys, respectively. Accuracy and response time (RT) were recorded for the search task. Each participant completed the study on their laptop or desktop computer. All images were displayed relative to the screen size to ensure that all targets and distractors always appeared at the same size. This was controlled using Python coding built into the experimental software. Probe images were equidistantly spaced in the search arrays. Probes were displayed at the same size as target preview faces.

### Design and procedure

A 3 (Group: Typical versus Distinctive versus Mixed) × 2 (Trial: Present versus Absent) mixed factorial design, repeated over the Trial factor, was used to examine performance in the DDTFS task.

Participants were sent (via email) information sheets and consent forms, which were signed electronically and returned to the experimenter before beginning the study. Participants then met the experimenter in the pre-experiment meeting held over Zoom Video Communications, Inc (2020 - zoom.us version 2.0.0). The meeting allowed the dissemination of best practice guidelines for completing the online tasks. Demographic information and verbal consent were collected.

All three tasks (CFMT + U, CFMT + I, and DDTFS) were completed in a single experimental session with short breaks allowed between each task. All participants completed the DDTFS task, followed by the CFMT + U and finally the CFMT + I.

#### DDTFS task

**Fig. 2:**
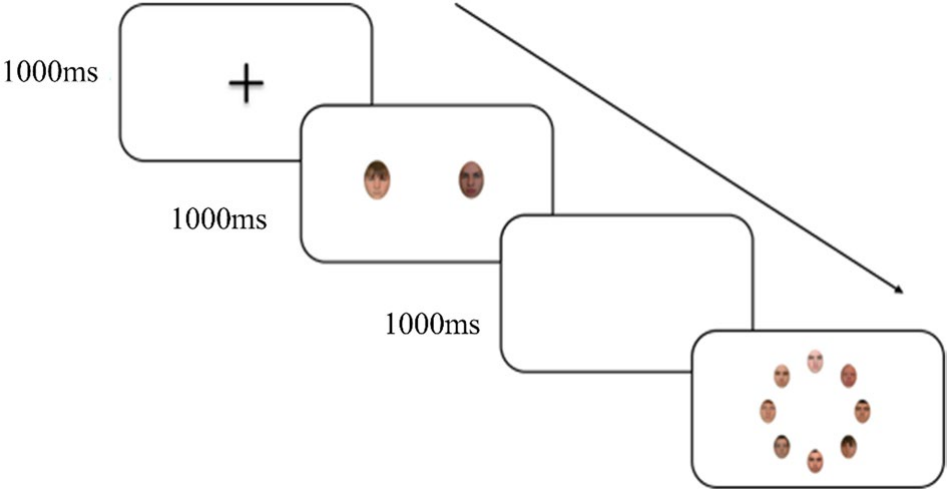
Image showing standard trial procedure for the dual-target search task

Participants were asked to search and respond if one of their target faces were present or both were absent. Group was a between-subjects factor, so participants searched for either two distinctive targets, two typical targets, or a typical and distinctive target. Trials started with a 1000 ms fixation cross followed by two simultaneously presented target preview images shown for 1000 ms. A 1000 ms blank screen followed the showing of the preview images before a search array containing eight equidistantly arranged faces positioned on a virtual circle (Fig. 2). Participants were instructed to respond both quickly and accurately, and the search arrays remained on the screen until a response was made. Incorrect responses were followed by a feedback noise to alert the participant to the error. The trial order was randomised across participants.

After completing all the search trials participants were asked to also make complete three similarity judgements. Participants were shown both of their target faces separately on screen and asked to rate how similar each was to the average face. They were then shown their two target faces on screen simultaneously and rated how similar they were to each other. All similarity judgements were on a 7-point Likert scale (1 [not similar] to 7 [very similar]). Responses for the ratings were made using a mouse to move a pointer to the desired value on the on-screen slider. Participants confirmed their ratings before finishing the search block of trials.^1^

#### *CFMT* + *tasks*

The CFMT + U and CFMT + I followed the standard procedure of Duchaine and Nakayama (2006) and expanded to the long-form variation (Russell et al., 2009).

Participants received debrief information after the completion of the study.

## Results

Data from four participants were removed from the analysis as accuracy in the CFMT + U task fell 2SD below the mean (scores equal to or less than 47; see Bowles et al., 2009). Two further participants were removed due to poor overall accuracy (2SD below the group mean for search accuracy in dual-target search trials) in the search task, resulting in a total of 102 participants’ data being included in the analysis. Trials with RTs < 500 ms or above 3SD from the group mean were removed (1.36% of data). Results for RT data are reported for correct trials, leaving 22,592 (92.28%) trials included in the analysis. Generalised eta-squared is reported as a measure of effect size (Bakeman, 2005) for ANOVAs. Bonferroni-corrected between-participant t tests were performed to explore the effect of distinctiveness, while Bonferroni-corrected within-participant t tests explored the effect of Trial type.

### Disjunctive dual-target face search

Accuracy (proportion correct) and RT data were analysed in a 3 (Group: Distinctive versus Typical versus Mixed) × 2 (Trial type: Present versus Absent) mixed factorial ANOVAs, repeated over the Trial type factor. In addition, we also computed signal detection measures (sensitivity (d’) and bias (c)) and conducted further analyses on these data. For the bias data, a positive value represents a conservative bias (tendency to respond ‘absent’), while a negative value represents a liberal bias (tendency to respond ‘present’).

### Accuracy

To explore whether performance for any Group performance was at ceiling separate, one-tailed one-sample t tests were conducted for overall task performance. In all conditions, performance accuracy significantly differed from ceiling (Distinctive *t*(35) = 7.57, *p* < 0.001, *d* = 1.26; Mixed t(31) = 8.1, *p* < 0.001, *d* = 1.43; Typical t(33) = 8.57, *p* < 0.001, *d* = 1.47).

**Fig. 3:**
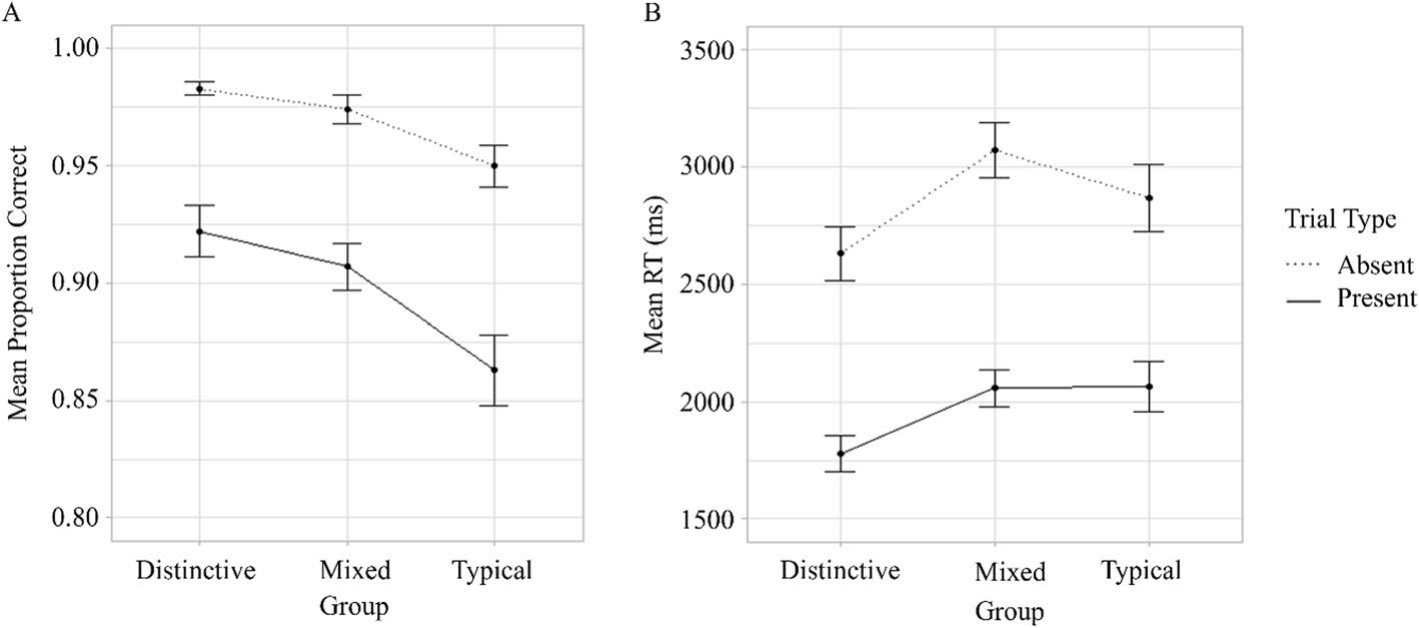
Mean proportion correct (panel A) and response times (panel B) for different groups on target-present and target-absent trials in Experiment 1. *Note* Error bars show standard error

The main effects of Group (*F(*2, 99) = 8.24,* p* < 0.001*,* $\eta_{G}^{2}$ = 0.112) and Trial type (*F(*1, 99) = 168.22*, p* < 0.001, $\eta_{G}^{2}$ = 0.294) were significant. Participants were more accurate in the Distinctive and Mixed groups than in the Typical group (*M* = 0.95, *SE* < 0.01, *M* = 0.94, *SE* < 0.01 versus *M* = 0.91, *SE* = 0.01, *p* < 0.001 and *p* = 0.011, respectively). The difference in performance accuracy between the Distinctive and Mixed groups did not reach significance (*p* = 0.945). Accuracy was higher on target-absent (*M* = 0.97, *SE* < 0.01 versus *M* = 0.90, *SE* < 0.01, *p* < 0.001) than on target-present trials. The interaction between Group and Trial did not reach significance (*F*(2,99) = 2.09*, p* = 0.130*,* $\eta_{G}^{2}$ = 0.01, Fig. 3).

### Response time

The main effects of Group (*F*(2,99) = 3.53, *p* = 0.033, $\eta_{G}^{2}$ = 0.06) and Trial type (*F*(1,99) = 1328.71, *p* < 0.001, $\eta_{G}^{2}$ = 0.43) were significant. Responses were faster in the Distinctive than Mixed (*M* = 2205.60, SE = 85.31 versus *M* = 2564.98, SE = 95.35, respectively, *p* = 0.019) groups. The difference in RTs between the Distinctive and Typical (*M* = 2466.81, SE = 102.14,* p* = 0.337) groups and Mixed and Typical groups (*p* = 0.733) did not reach significance. Responses were faster on target-present than target-absent trials (*M* = 1961.73, SE = 53.15 versus *M* = 2849.10, SE = 74.31, respectively, *p* < 0.001). The interaction between Group and Trial did not reach significance (*F*(2,99) = 2.88, *p* = 0.061, $\eta_{G}^{2}$ = 0.003, Fig. 3).

These initial analyses demonstrate two key findings, first that search performance is more accurate when at least one of the faces in the pairing is distinctive, such that search for two typical faces is relatively difficult. Second, relative to the distinctive group, there is a cost to maintaining accurate DDTFS in the Mixed group that is reflected in response times.

### Signal detection analysis

Following the protocol set out by Macmillan & Creelman (2005), the accuracy data were used to compute signal detection measures of sensitivity (*d’*) and bias (c). Sensitivity and bias were analysed separately in one-way between subject ANOVAs comparing the effect of Group.

### Sensitivity (d’)

**Fig. 4:**
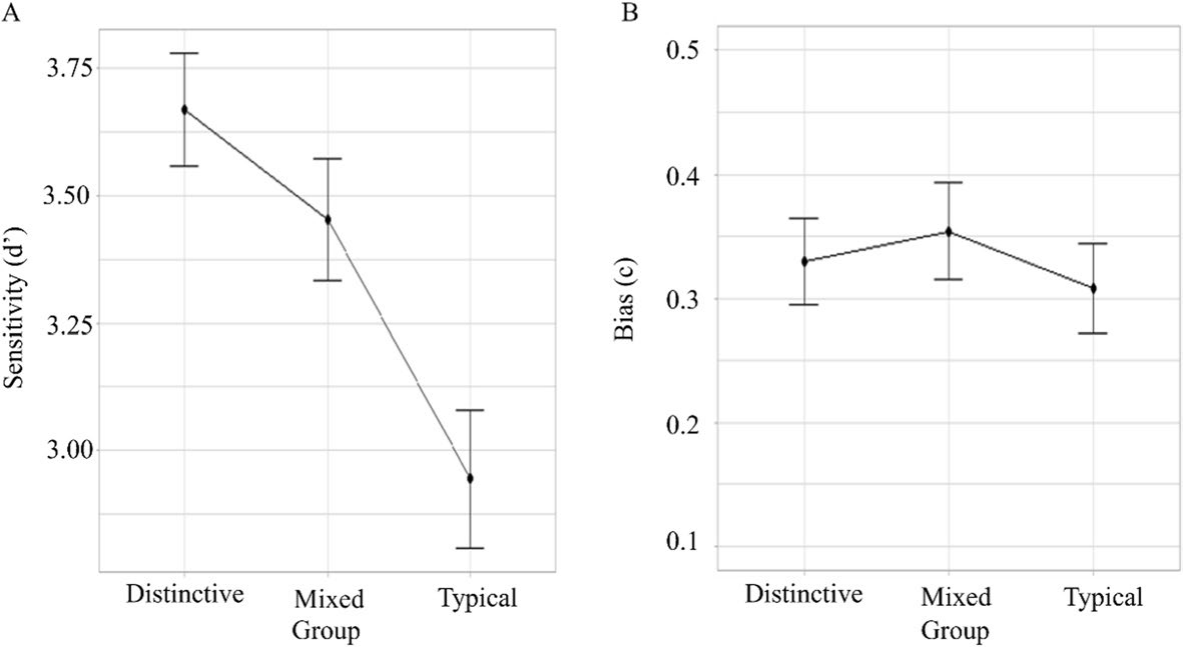
Mean sensitivity (panel A) and bias (panel B) for different groups in Experiment 1. *Note* Error bars show standard error

The main effect of Group (*F*(2,99) = 9.47, *p* < 0.001, $\eta_{G}^{2}$ = 0.161) was significant (Fig. 4). The difference in sensitivity for Distinctive (*M* = 3.67, SE = 0.11) and Typical (*M* = 2.95, SE = 0.14, *p* < 0.001) and Mixed (*M* = 3.45, SE = 0.12) and Typical (*p* = 0.014) was significant. Participants were less likely to detect Typical faces than Mixed or Distinctive. The difference between the Distinctive and Mixed groups did not reach significance (*p* = 0.648).

### *Bias* (c)

There main effect of Bias did not reach significance (*F*(2,99) = 0.379, *p* = 0.686, $\eta_{G}^{2}$ = 0.008).

### Target prioritisation in DDTFS

The target with the higher accuracy was deemed, post hoc, to be prioritised (and the target with lower accuracy as non-prioritised).^2^ Prioritisation analysis was conducted on target-present trials only (as prioritisation cannot be assigned on target-absent trials). In a 3 (Group: Distinctive versus Typical versus Mixed) × 2 (Prioritisation: Prioritised versus Non-prioritised) mixed factorial ANOVA, repeated over the Prioritisation factor. As the main effect of Prioritisation is inevitable, and the main effect of Group has been reported above, the sole focus of interest here is on the interaction between Group and Prioritisation.^3^

### Accuracy

**Fig. 5:**
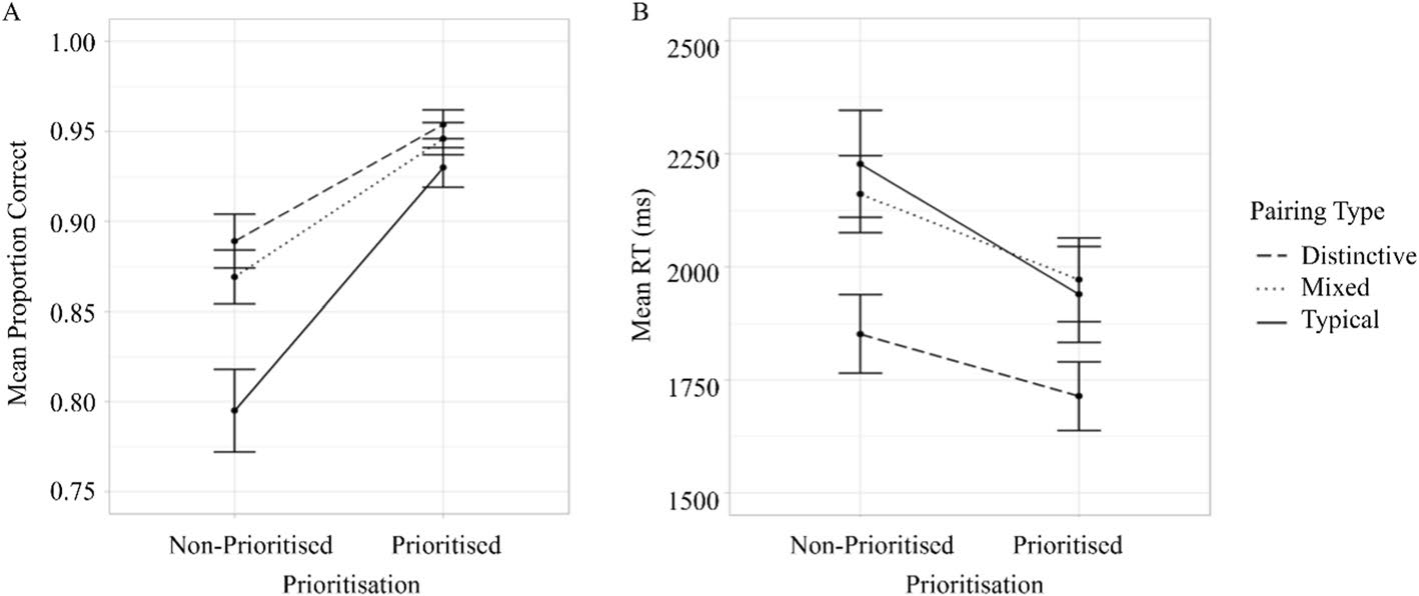
Mean proportion correct (panel A) and response times (panel B) for prioritised and non-prioritised targets for different groups in Experiment 1. *Note* Error bars show standard error

The interaction between Group and Prioritisation (*F*(2,99) = 5.62, *p* = 0.005, $\eta_{G}^{2}$ = 0.03, Fig. 5) was significant. Separate one-way between-participants ANOVAs were conducted for Group at each level of Prioritisation to break down the interaction. The effect of Group was significant for the Non-prioritised targets (*F*(2,99) = 7.57, *p* < 0.001, $\eta_{G}^{2}$ = 0.13) but not for the Prioritised targets (*F*(2, 99) = 1.75, *p* = 0.179, $\eta_{G}^{2}$ = 0.03). Non-prioritised targets in the Distinctive and Mixed groups were searched for more accurately than in Typical group (*p* < 0.001, *p* = 0.017, respectively). The difference in accuracy for Non-prioritised targets in the Distinctive and Mixed groups was not significant (*p* = 1).

### Response time

The interaction between Group and Prioritisation did not reach significance (*F*(2,99) = 1.68, *p* = 0.192, $\eta_{G}^{2}$ = 0.003, Fig. 5).

Overall, the prioritisation analysis shows that Non-prioritised faces in the Typical group were searched for less accurately than in the Distinctive and Mixed groups.

### *The association between CFMT* + *and DDTFS*

Correlations were computed using Pearson’s *r* for arcsine-transformed accuracy and log-transformed RTs from ‘correct’ responses and with both versions of the CFMT+. All tests of significance for correlations were two-tailed. Bonferroni correction for multiple was applied, the significant alpha level was set at 0.017 (0.05/3 -the number of experimental groups), and 95% confidence intervals were reported. Scores on the CFMT + U and CFMT + I ranged from 51 to 96 out of 102 (*M* = 76.77, SD = 11.28) and 33 to 84 out of 102 (*M* = 57.54, SD = 10.92), respectively.

### Prioritised target

No correlations between CFMT + and search accuracy reached significance (Table 1). The correlations between CFMT + U and CFMT + I and RTs were significant for the Typical group (accounting for 26.01% and 19.36% of the variance) but did not reach significance for any other groups.

**Table 1:** Correlations with confidence intervals for all groups and the CFMT + in Experiment 1

	Prioritised			Non-Prioritised		
	Distinctive	Typical	Mixed	Distinctive	Typical	Mixed
*Accuracy*						
CFMT + U	0.10	− 0.066	− 0.035	0.238	0.427*	0.346
	[− 0.23, 0.42]	[− 0.40, 0.28]	[− 0.38, 0.32]	[− 0.10, 0.53]	[0.10, 0.53]	[− 0.003, 0.62]
CFMT + I	0.123	0.102	0.155	0.198	0.512 *	0.389
	[− 0.21, 0.43]	[− 0.25, 0.43]	[− 0.21, 0.48]	[− 0.14, 0.49]	[0.21, 0.73]	[0.05, 0.65]
*RT*						
CFMT + U	− 0.105	− 0.514*	0.268	− 0.218	− 0.580*	0.089
	[− 0.42, 0.23]	[− 0.73, − 0.21]	[− 0.09, 0.56]	[− 0.51, 0.12]	[− 0.77, 0.30]	[− 0.27, 0.42]
CFMT + I	− 0.017	− 0.443*	0.125	0.028	− 0.448 *	0.012
	[− 0.34, 0.31]	[− 0.68, − 0.12]	[− 0.23, 0.45]	[− 0.30, 0.35]	[− 0.68, − 0.13]	[− 0.34, 0.40]

Values in the square brackets indicate the 95% confidence intervals for each correlation

*Indicates *p* < 0.017

### Non-prioritised target

The correlations between CFMT + U and CFMT + I and accuracy were significant for the Typical group (accounting for 18.49% and 26.01% of the variance, respectively) but did not reach significance for any other group. The correlations between CFMT + U and CFMT + I and RTs were significant in the Typical group (accounting for 33.64% and 20.25% of the variance, respectively).

Overall, the speed of RTs to Prioritised and Non-prioritised faces and the accuracy of the search for Non-prioritised faces was associated with scores on both the CFMT + U and CFMT + I but only for the Typical group.

## Discussion

Experiment 1 explored the role of target distinctiveness in determining the accuracy and speed of search for targets in the DDTFS task. The results found no effect of group on the accuracy of the search for Prioritised targets but a marked one on Non-prioritised targets. Non-prioritised faces in the Distinctive and Mixed groups were searched for more accurately than in the Typical condition. It is the search for more than one typical face that leads to errors in DDTFS for unfamiliar faces. The signal detection d’ analysis confirmed that the difference in accuracy across conditions results from the task conditions leading to a change in sensitivity rather than criterion. The ability to conduct DDTFS with typical faces was equally associated with performance on the CFMT + when the tasks were performed upright and inverted. We reasoned in the Introduction that such a finding would be consistent with face search on the basis of featural information present in faces as opposed to holistic information. We return to consider this issue in the General Discussion.

We interpret the results as consistent with distinctive targets being encoded into long-term memory more quickly than typical faces, thus allowing them to draw on long-term memory (Wolfe, 2021). In contrast, when both targets are typical, they draw on WM for longer, requiring one face to be prioritised to help manage resources. The task of encoding pairs of typical faces leads to increased reliance on WM and marked associations with performance on the CFMT+, especially with respect to non-prioritised faces.

The results have implications for the findings of Mestry et al. (2017). Mestry et al. reported a general difficulty when searching for two faces relative to searching for one. With respect to accuracy, the present data show this holds when both faces are typical but not otherwise. With respect to RTs, responses in the Distinctive group were faster than in the Mixed or Typical groups. The conclusion we reach is that DDTFS is difficult when both faces are typical and relatively easy when both faces are distinctive. When search is for one face that is distinctive and is the other typical, participants are accurate but take a long time to reject a trial as not containing a target. The present findings are consistent with those of Mestry et al. but show a limit to which their results generalise.

Experiment 1 allowed participants to encode target faces over search trials. This aspect of the design means that we cannot distinguish between the effects of distinctiveness per se and effects of distinctiveness that are mediated by face encoding. In Experiment 2, we repeat Experiment 1 but precede the DDTFS with two single-target search blocks. The question is whether distinctiveness continues to exert an influence on the speed of DDTFS even when faces are well-encoded.

## Experiment 2

### Method

All details were the same as in Experiment 1 except for those specifications outlined below.

### Participants

The effect size reported in Experiment 1 ($\eta_{G}^{2}$ = 0.112) was used to estimate the sample size. This was converted to a Cohen’s *F* = 0.36 which for an alpha = 0.05 and power = 0.95 gave a required sample size of 24 to observe the effect. For completeness, we estimated the main effect of trial type using the effect size reported in Experiment 1 ($\eta_{G}^{2 }$ = 0.294) converted to Cohen’s *F* = 0.64, requiring a sample size of 9 to observe the effect. As the interaction in Experiment 1 was not significant, we again estimated the sample size using a small-medium effect (*F* = 0.15) which required 120 participants to achieve 0.95 power and 78 to achieve a power = 0.8 to observe the interaction. For the effect of single-target vs dual-target search, we again estimated the sample size from the effect size reported by Mestry et al. (2017). Mestry et al. reported an effect size of $\eta_{G}^{2}$ = 0.121 for the number of targets. This was again converted into Cohen’s *F* = 0.9 requiring a sample size of 6 to observe the effect.

A total of 108 new participants were recruited and pseudo-randomly allocated into one of three groups (*n* = 36 per group). Participants ranged in age between 18 and 59 years (*M* = 21.17, SD = 4.83), 20 participants were male, one participant self-reported as gender fluid, and a second preferred not to say. The remaining participants identified as female. Participants were recruited via the Liverpool Hope and Bournemouth University participation schemes. Students received course credits for their participation. All participants reported normal colour vision and normal or corrected to normal visual acuity. Full ethical approval was granted from Liverpool Hope University.

### Design and procedure

Participants completed a block of ten practice trials, and three blocks of experimental trials: two blocks (one for each target) of 120 trials of single-target face search followed by a block of 240 trials of dual-target face search. Group (Distinctive versus Typical versus Mixed) was a between-participant factor and the Number of targets (Single versus Dual) and Trial type (Present versus Absent) were within-subject factors. Participants completed all three blocks of trials in one of the three groups. The order of targets in the single-target search was counterbalanced. All other details remained as in Experiment 1.

## Results

Data and results were treated as in Experiment 1. As in Experiment 1, Bonferroni-corrected between-participant t tests were performed for pairwise comparisons exploring the effect of distinctiveness, while Bonferroni-corrected within-participant t tests were used to examine the effects of Trial type and Number of targets. Two participants were removed from the analysis as accuracy in the CFMT + U task fell 2SD below the mean and four due to low accuracy (< 2SD below the group mean for search accuracy in dual-target search trials) in the search task. As a result of these exclusions, 102 participant’s sets of data were included in the analysis. 1.16% of single-target search trials and 1.45% of DDTFS trials were removed from the data for RTs being < 500 ms or > 3SD from the group mean. In addition, results are reported for correct trials only therefore 5.48% of single-target trials and 5.96% of dual-target trials were removed due to participants making incorrect responses. For single-target trials a total of 22,871 trials and for dual-target a total of 22,688 trials were analysed.

### Disjunctive dual-target face search

#### Accuracy

To explore whether the performance for any Group was at ceiling separate, one-tailed one-sample t tests were conducted for overall task performance. In all conditions, the performance accuracy significantly differed from ceiling (Distinctive *t*(33) = 5.20, *p* < 0.001, *d* = 0.89; Mixed *t*(33) = 4.81, *p* < 0.001, *d* = 0.82; Typical *t*(33) = 6.94, *p* < 0.001, *d* = 1.19).

The main effect of Trial type (*F*(1,99) = 106.13, *p* < 0.001, $\eta_{G}^{2}$ = 0.21) was significant. The search was more accurate in target-absent trials than in target-present trials (*M* = 0.98, SE < 0.01 versus *M* = 0.90, SE < 0.01, respectively, *p* < 0.001). The main effects of Group (Distinctive *M* = 0.94, SE < 0.01; Mixed* M* = 0.93, SE < 0.01; Typical* M* = 0.95, SE < 0.01) and Number of targets (Single *M* = 0.94, SE < 0.01; Dual *M* = 0.94, SE < 0.01) did not reach significance (*F* < 1, *F*(1,99) = 1.14, *p* = 0.288, $\eta_{G}^{2}$ = 0.002, respectively).

**Fig. 6:**
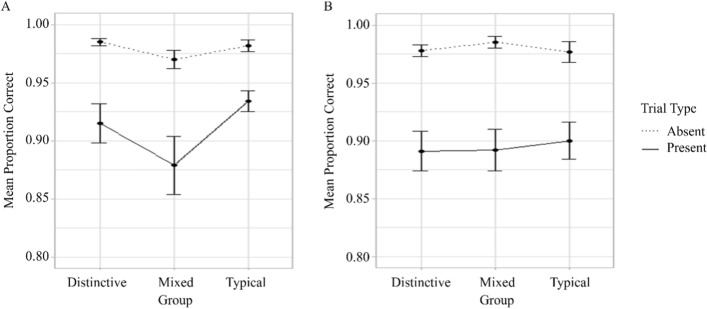
Mean proportion correct for different groups on target-present and target-absent trials in single (panel A)- and dual-target (panel B) search Experiment 2. *Note* Error bars show standard error

None of the two-way interactions between Group and Number of targets (*F*(2,99) = 3.01, *p* = 0.054, $\eta_{G}^{2}$ = 0.01), Group and Trial type (*F*(2,99) = 1.24, *p* = 0.293, $\eta_{G}^{2}$ = 0.006), Number of targets and Trial type (*F*(1,99) = 3.86, *p* = 0.052, $\eta_{G}^{2}$ = 0.003) nor the three-way interaction between Group, Number of targets and Trial type (*F*(2,99) = 0.91, *p* = 0.406, $\eta_{G}^{2}$ = 0.001) reached significance (Fig. 6).

#### Response time

The main effects of the Number of targets (*F*(1,99) = 24.91,* p* < 0.001, $\eta_{G}^{2}$ = 0.03) and Trial type (*F*(1,99) = 1334.75, *p* < 0.001, $\eta_{G}^{2}$ = 0.44) were significant. RTs were shorter for single-target search than for DDTFS trials (*M* = 1933.74, SE = 43.73 versus *M* = 2062.77, SE = 44.96, respectively, *p* < 0.001) and on target-present trials than target-absent trials (*M* = 1619.50, SE = 28.57 versus *M* = 2377.01, SE = 41.77, respectively, *p* < 0.001). The main effect of Group (Distinctive *M* = 1925.25, SE = 52.74; Mixed *M* = 2144.22, SE = 56.91; Typical *M* = 1925.30, SE = 52.04) did not reach significance (*F*(2,99) = 2.25, *p* = 0.111, $\eta_{G}^{2}$ = 0.04).

**Fig. 7:**
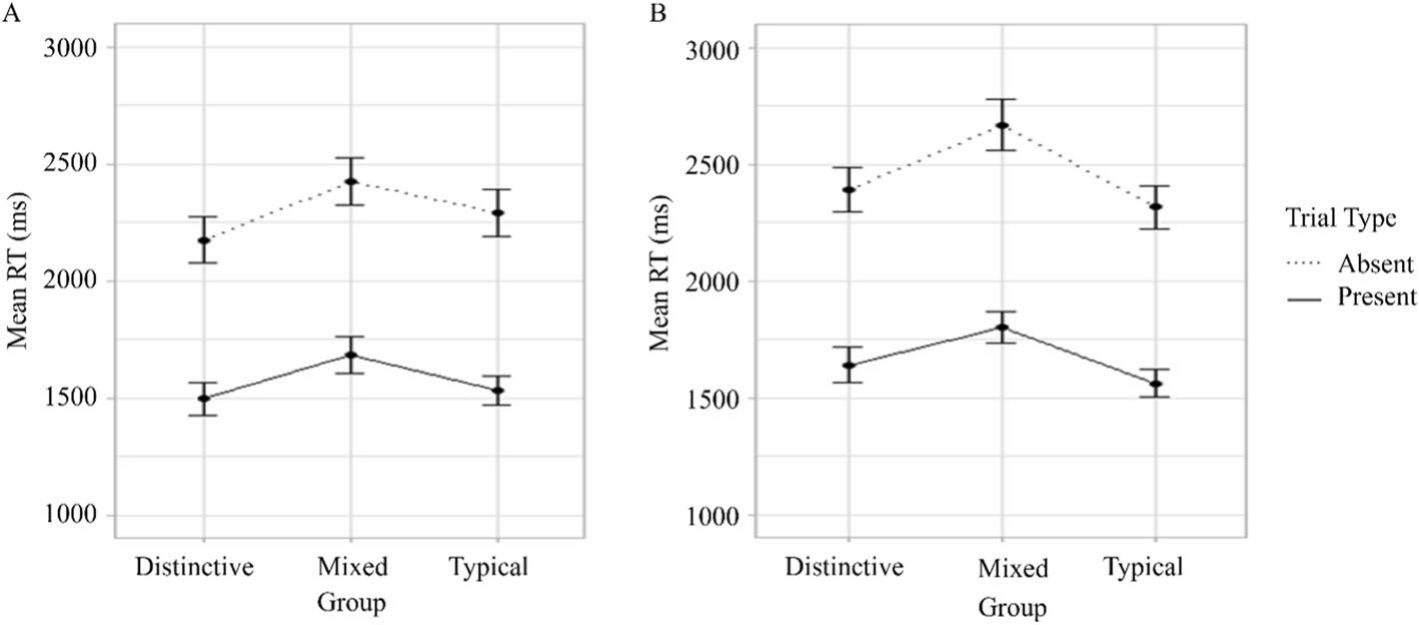
Mean response times for different groups on target-present and target-absent trials in single (panel A) and dual-target (panel B) search Experiment 2. *Note* Error bars show standards error

The two-way interactions between Group and Number of targets (*F*(2,99) = 2.35, *p* = 0.101, $\eta_{G}^{2}$ = 0.01), Group and Trial type (*F* < 1) and between Number of targets and Trial type (*F*(1,99) = 2.51, *p* = 0.116, $\eta_{G}^{2}$ = 0.001) and the three-way interaction between Group, Trial type and Number of targets were all non-significant (*F* < 1, Fig. 7).

Overall, the analysis of accuracy and RT data showed no effect of Group. As expected, absent trials were responded to more accurately and slower than present trials and the DDTFS task was responded to more slowly than the single-target search.

### Signal detection analysis

#### Sensitivity (d’)

The main effect of Group (*F*(2,99) = 2.064,* p* = 0.132, $\eta_{G}^{2}$ = 0.04) failed to reach significance.

#### *Bias* (c)

The main effect of Group failed to reach significance (*F*(2,99) = 1.13, *p* = 0.327, $\eta_{G}^{2}$ = 0.022).

#### Target prioritisation in DDTFS

Group did not influence overall accuracy or RT in Experiment 2; nevertheless, the target-present trials from the dual-target condition were split into prioritised and non-prioritised targets and analysed as in Experiment 1.^4^

#### Accuracy

**Fig. 8:**
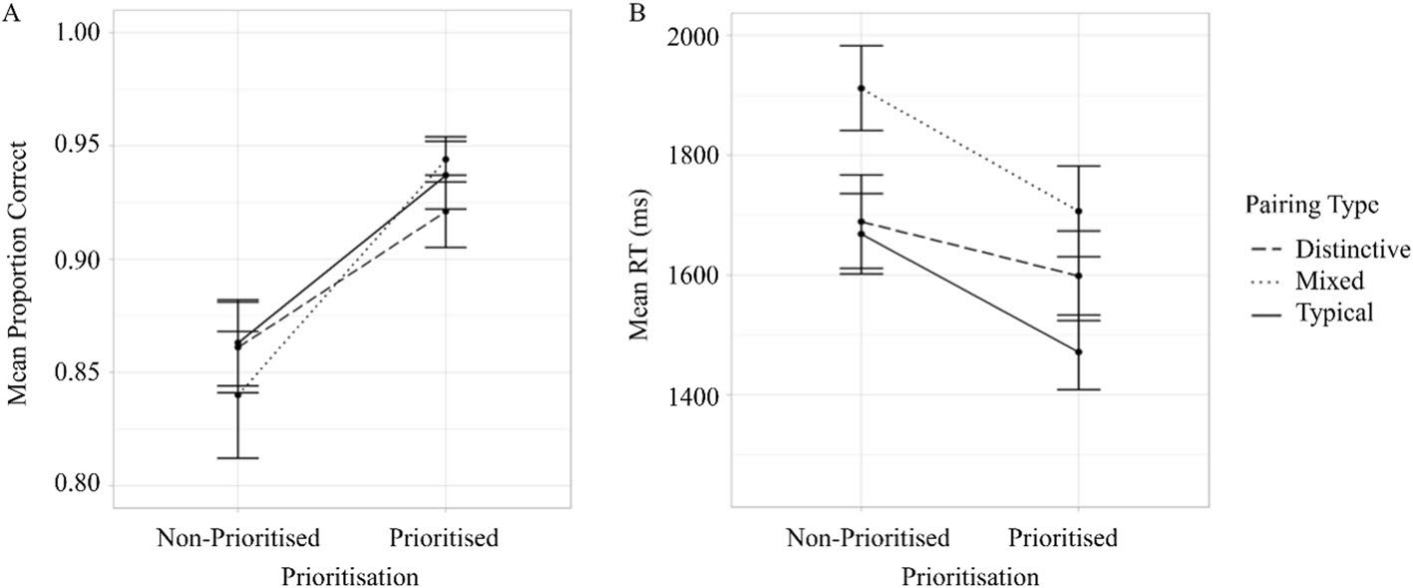
Mean proportion correct (panel A) and response times (panel B) for prioritised and non-prioritised targets in different groups in Experiment 2. *Note* Error bars show standards error

The interaction between Group and Prioritisation did not reach significance (*F*(2,99) = 2.02, *p* = 0.138, $\eta_{G}^{2}$ = 0.007, Fig. 8).

#### Response time

The interaction between Group and Prioritisation did not reach significance (*F*(2,99) = 2.27, *p* = 0.11, $\eta_{G}^{2}$ = 0.01, Fig. 8).

In sum, and in contrast to Experiment 1, there was no evidence of an interaction between Group and Prioritisation in either analysis of accuracy or response times.

#### *The association between CFMT* + *and the DDTFS*

The scores on the CFMT + U and CFMT + I ranged from 48 to 97 out of 102 (*M* = 73.61, SD = 12.27) and 32 to 86 out of 102 (*M* = 56.07, SD = 10.25), respectively.

#### Prioritised targets

There are no correlations between the CFMT + U or CFMT + I, and search accuracy reached significance. Only the correlation between scores on the CFMT + I and RT for the Mixed group was significant and explained 18.49% of the variance (Table 2).

**Table 2:** Correlations with confidence intervals for all pairing types and the CFMT + in Experiment 2

	Prioritised			Non-Prioritised		
	Distinctive	Typical	Mixed	Distinctive	Typical	Mixed
*Accuracy*						
CFMT + U	− 0.036	0.248	0.247	0.058	0.351	0.404
	[− 0.37, 0.31]	[− 0.10, 0.54]	[− 0.10, 0.54]	[− 0.29, 0.39]	[0.02, 0.62]	[0.08, 0.65]
CFMT + I	− 0.059	0.079	0.343	− 0.001	0.198	0.496*
	[− 0.39, 0.29]	[− 0.27, 0.41]	[0.01, 0.61]	[− 0.34, 0.34]	[− 0.15, 0.50]	[0.12, 0.68]
*RT*						
CFMT + U	0.095	0.230	− 0.293	− 0.147	− 0.002	− 0.247
	[− 0.25, 0.42]	[− 0.12, 0.53]	[− 0.57, 0.05]	[− 0.44, 0.21]	[− 0.34, 0.34]	[− 0.54, 0.10]
CFMT + I	− 0.152	.0.019	− 0.432*	− 0.350	− 0.209	0.050
	[− 0.47, 0.20]	[− 0.32, − 0.36]	[− 0.67, 0.11]	[− 0.61, − 0.01]	[− 0.51, 0.14]	[− 0.30, 0.39]

Values in the square brackets indicate the 95% confidence intervals for each correlation

*Indicates *p* < 0.017

#### Non-prioritised targets

No correlations between the CFMT + U score and search accuracy reached significance. Search accuracy did correlate with the CFMT + I score when searching in the Mixed group (accounting for 19.36% of the variance). No correlations between the CFMT + I and Distinctive or Typical groups reached significance. No correlations between CFMT + U and CFMT + I scores and RT reached significance.

In sum, there are only associations between the speed of search for prioritised faces, and the accuracy of search for non-prioritised faces in the Mixed group and performance in the inverted CFMT + task reached significance.

#### Comparison of Experiments 1 and 2.

**Fig. 9:**
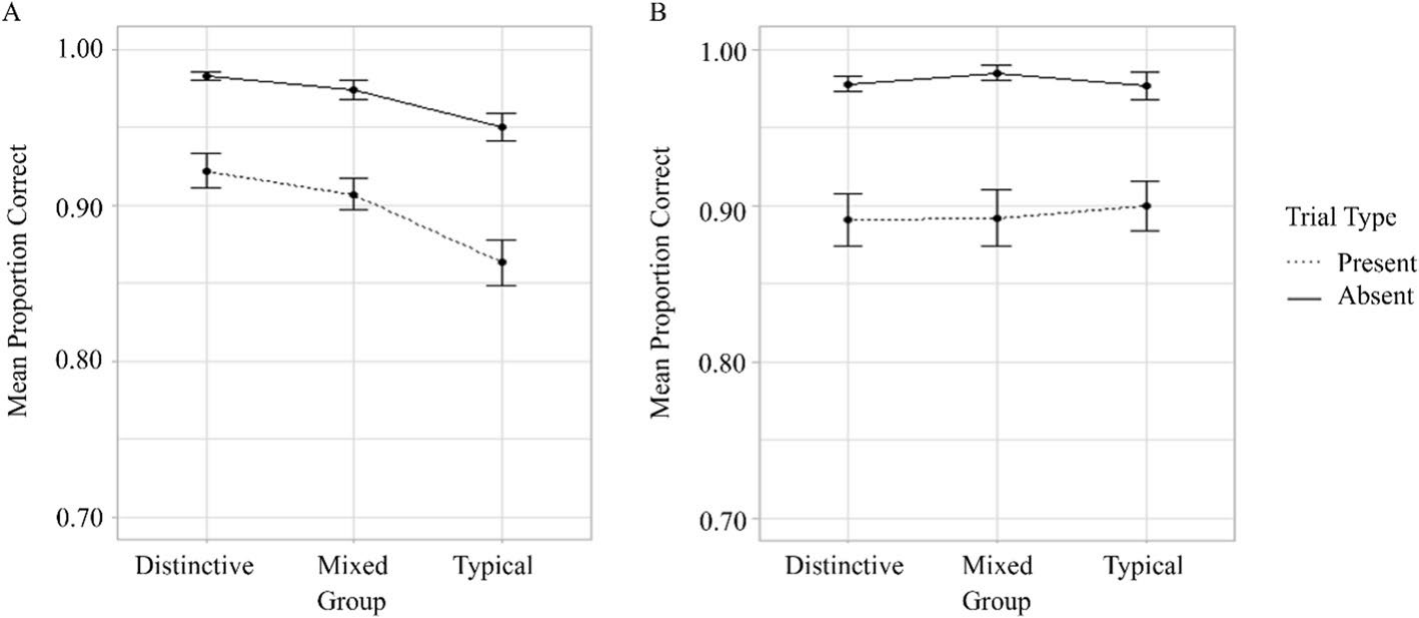
Mean proportion correct for different groups in target-present and target-absent dual-target search trials in Experiment 1 (panel A) and Experiment 2 (panel B). *Note* Error bars show standard error

**Fig. 10:**
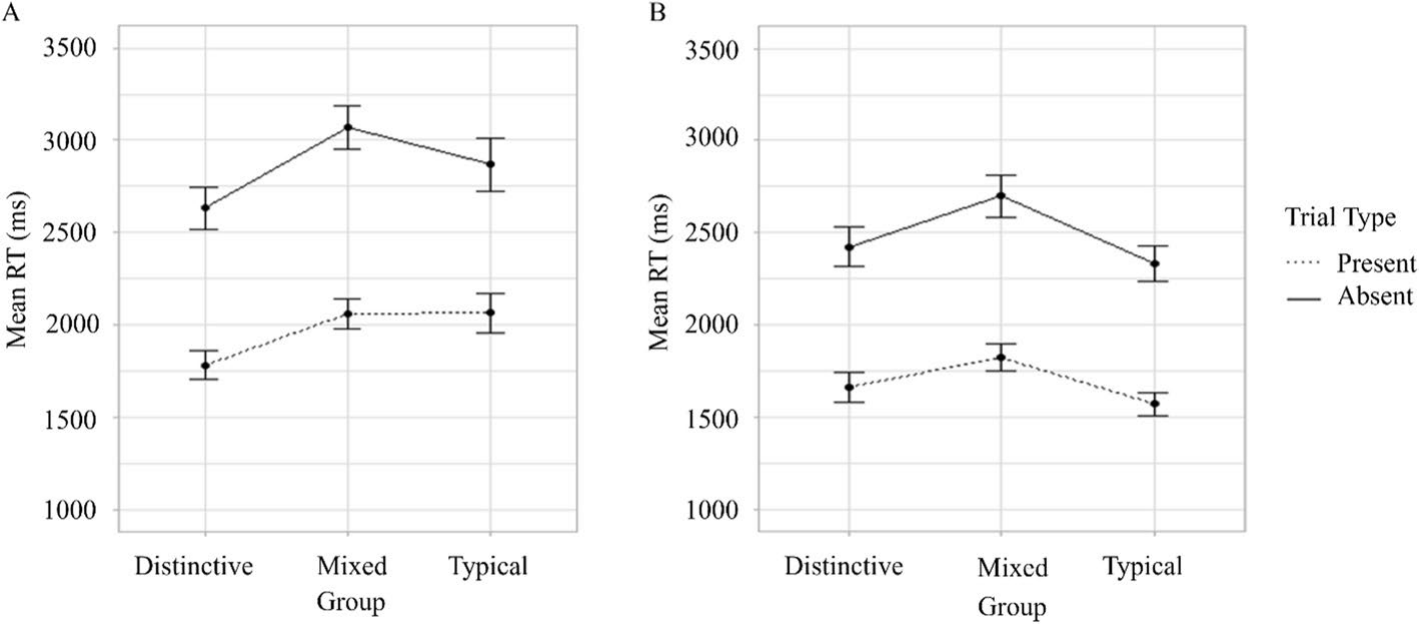
Mean response times for different groups on target-present and target-absent dual-target search trials in Experiment 1 (panel A) and Experiment 2 (panel B). *Note* Error bars show standard error

Finally, the accuracy and response times from the dual-target search trials of Experiments 1 and 2 were compared directly using a 3 (Group: Distinctive versus Mixed versus Typical) × 2 (Trial type: Present versus Absence) × 2 (Experiment: Experiment 1 versus Experiment 2) mixed factorial ANOVAs. The critical finding from the accuracy analysis was an interaction between Experiment and Group (*F*(2,198) = 3.69, *p* = 0.027, $\eta_{G}^{2}$ = 0.03; see Fig. 9). The effect of Experiment was significant for the Typical (*p* = 0.025) group but not for the Mixed (*p* = 0.879) or Distinctive (*p* = 0.147) groups. With respect to response times, these were faster in Experiment 2 than 1 (*F*(1,198) = 18.434,* p* < 0.001, $\eta_{G}^{2}$ = 0.08, Fig. 10). No other interactions involving Experiment were significant in either analysis (*p* > 0.05).

## Discussion

Experiment 2 explored the role of the relative distinctiveness of faces in DDTFS when participants are first given the opportunity to encode faces by virtue of completing separate single-target searches for each target. The results of Experiment 2 show two key findings. First, the effect of Group was negligible such that the influence of target typicality on performance was very limited. Specifically, Group has no influence on search accuracy and only a very limited influence on response times. (RTs were [at least numerically] slower in the Mixed group compared to the other groups and associated with scores on the CFMT + I.) Second, while the DDTFS was performed more slowly than the preceding single-target search blocks, it was not performed less accurately.

The cross-experiment analysis confirms the effect of allowing participants to complete two blocks of single-target search with the target faces before completing the DDTFS task was to remove the group differences reporting in Experiment 1. These results suggest that the findings of Experiment 1 reflect the effects of distinctiveness on encoding of unfamiliar faces on dual-target face search. The findings from Experiment 2 adds to those of Experiment 1 in that, even when apparently well-encoded in single-target search, there may still be a slowing in the search for the non-prioritised target if the pair of targets differs in distinctiveness (as they do for the Mixed group). The magnitude of the difficulty is associated with scores on the CFMT + I. We will return to discuss these findings in the General Discussion.

While not designed to test the difficulty of the dual-target face relative to single-target search, the results of Experiment 2 are nevertheless consistent with the idea that dual-target face search is slower than single-target face search.

## General discussion

The present study explored the role of facial distinctiveness in determining the accuracy and speed of target detection in DDTFS for unfamiliar targets. The results of Experiment 1 showed that targets were missed if both faces were typical. Importantly, misses were not shared equally across the targets. Misses were focussed on one of the target faces: we refer to this effect as target shedding. The need to shed a typical target disappeared in Experiment 2 when participants were able to first learn the face targets in single-target blocks before completing the dual-target block. We conclude that the difficulty of performing DDTFS in the typical group in Experiment 1 is caused by the need to focus attention so that faces can be encoded into long-term memory. The contrast across groups is consistent with the encoding of typical faces being slowed relative to distinctive faces such that the disjunctive search for pairs of typical faces relies on WM for longer than the disjunctive search for pairs of distinctive faces.

The RT data did also lead to a further finding that was demonstrated in both Experiments 1 and 2. RTs were longer than might be expected in the Mixed group when searching for a typical and a distinctive face. Given that raised RTs were found in the Mixed group when faces were encoded during dual-target search (Experiment 1) and in single-target blocks (Experiment 2), the cause of the effect is puzzling. Our intuition is that establishing a threshold for determining recognition of distinctive faces is different from that required for typical faces (Valentine, 1991). Nevertheless, when the targets being sought are both typical or both distinctive, there is some certainty over the difficulty of making an identification judgement such that setting a decision threshold can be done with confidence. In the case of the Mixed group, however, uncertainty exists in the making of an identification decision and seems to be reflected in relatively long RTs being needed to support accurate search.

The issue of certainty over the ease of target recognition decisions might also be affected by the range of distinctiveness in the distractor faces. For example, if all distractors had been typical or all distinctive, then this might have influenced the ease of identification. It is important, therefore, to note that the distractors in Experiments 1 and 2 were randomly selected from the set of 120 faces that were not rated as either the most or least distinctive faces by participants taking part in the pre-study of Experiment 1 and so this issue is unlikely to have affected the results of the present study.

The primary purpose of the present study was not to compare dual- and single-target searches directly. Indeed Experiment 1 did not include single-target conditions and the order of the single- and dual-target conditions was not counterbalanced in Experiment 2. Nevertheless, once faces were encoded, RTs in the DDTFS task were longer than in the preceding single-target conditions. The results of Experiment 2 are, therefore, consistent with dual-target cost on the speed of face search (Mestry et al., 2017), even when faces are robustly encoded. However, it is important to emphasise that we are not saying training on single-target search is critical to understanding the differing results found across Experiments 1 and 2. For example, it might be that blocks of training with dual-target search might also be effective in removing the impact of prioritisation from the disjunctive search for pairs of typical faces. The comparison of the effectiveness of single- and dual-target training is for a future study to explore.

Participants completed the CFMT + in both upright and inverted conditions to see if correlations with the accuracy and speed of search for non-prioritised faces were higher with scores on the CFMT + U than the CFMT + I. If this were so, then we hypothesised in the Introduction that it would be consistent with some role for holistic processing in the accuracy and speed of search for non-prioritised faces. Correlations were found in both Experiment 1 (in the Typical condition) and 2 (in the Mixed condition). These findings are consistent with two conclusions. First, the magnitude of prioritisation in the Typical group and the ability to search for non-prioritised typical faces is associated with the score in the CFMT + (whether completed upright or inverted). We interpret this novel finding as evidence that the ability to perform accurate DDTFS for unfamiliar typical faces is associated with the ability to recognise faces. While it remains possible that holistic processing may impact search for typical faces in DDTFS, the pattern of association in Experiments 1 and 2 is most consistent with DDTFS being based on featural information present in faces rather than more holistic information. It is, however, an issue worthy of further investigation.

### Constraints on generality

We believe that the findings we report reflect a basic limitation in the search for unfamiliar faces. Nevertheless, it is very likely that the specific findings we report are influenced by the stimulus set and selection of participants. In this regard, it is right to emphasise that the stimuli were all white males, whereas the participants were largely white females. We think it likely that the effect of prioritisation found in the present study will be influenced by other factors affecting attentional selection and face encoding. The range of factors that influence attentional selection and prioritisation during face encoding might include at a minimum, age, sex, and ethnicity (Backman, 1991; Meissner & Brigham, 2001; Sporer, 2001; Wright et al., 2001). Indeed, we believe that the interaction of participant and stimulus identities is an important line of research to pursue in the future. In addition, the study was conducted using still photographs on a screen. As such, extrapolating the present findings to the real world of security search may well require caution.

It would be ideal to run search studies in a laboratory using a fixed experimental setup; however, this was not possible in the current study (as data were collected during the COVID-19 pandemic). This concern relates more closely to gathering and interpreting RT measures than accuracy data. However, recent exploration of the veracity of RTs gathered across devices, operating systems, and browsers shows that the data are generally reliable. In particular, so long as RTs exceed 100 ms, then the differences across devices are rather minimal (Anwyl-Irvine et al., 2020). In the current experiments, RT typically varied in a range from just under 1 s to around 3.5 s. Moreover, reliable (and predicted) differences and associations with the CFMT + were found in the analysis of the RT data. We suggest that repeating the experiments in the laboratory may strengthen the observed effects but would not change the nature of the effects we report here.

A further question worth exploring is if the tendency to prioritise faces might occur with factors beyond typicality. We have interpreted the need for prioritisation in terms of the differential demands placed in WM by the effect of typicality on facial encoding. An open question is if a similar effect might emerge through biases in attention to different kinds of faces. For example, implicit bias in resource allocation is brought about by ethnicity, attraction, or emotional valence. The present study is unable to answer these questions though they are important ones that should be explored in future work.

The main conclusions of the current study are that (1) distinctiveness, a factor that influences the rate of face learning, influenced the accuracy of DDTFS for unfamiliar faces; (2) the cost of DDTFS for unfamiliar faces were loaded onto one, non-prioritised, target such that accuracy on the other was good; and (3) the magnitude of the cost borne by the non-prioritised target was inversely related to face processing ability, as indexed by the CFMT+.

The results have three important consequences for those tasked with identifying unfamiliar faces in security situations in the real world. First, if more than one target is to be detected, then searchers must be given sufficient opportunity to encode target faces robustly. This is especially so if the target faces are rather typical in appearance. Second, if a job requires searchers to perform a disjunctive face search task, then careful assessment of the tendency for a searcher to prioritise faces should be made. Third, given the choice, in situations where searchers are likely to need to be on the lookout for more than one face, it would be better to select for employment those scoring highly on the CFMT + as the tendency to prioritise faces will be minimised.

In conclusion, searching for more than one unfamiliar face is difficult especially if both faces are typical. In the case of typical face pairs, one face is initially prioritised over the other. With practice, pairs of typical faces can be searched for accurately. However, there remains a cost to RT relative when searching for pairs of faces relative to single faces. Better performance on the CFMT + is associated with the speed and accuracy of the DDTFS when a search is most challenging.

### Open practices statement

All data, analysis code and research materials have been made publicly available on the Open Science Framework and can be accessed at https://osf.io/4pc9y/?view_only=18e05622272548319cb6280cb3d52922. The study was not preregistered. Data were analysed using R, version 4.1.3 (R Core Team, 2021), and the package *ggplot*, version 3.3.5 (Wickham, 2016). This study’s design and its analysis were not preregistered. We report how we determined our sample size, all data exclusions, all manipulations, and all measures in the study, and we follow JARS (Kazak, 2018).

## Data Availability

The datasets generated and/or analysed during the current study are available in the Open Science Framework repository, https://osf.io/4pc9y/?view_only=18e05622272548319cb6280cb3d52922.

## Abbreviations

CFMT+: Cambridge Face Memory Test
CFMT + U: Cambridge Face Memory Test Upright Version
CFMT + I: Cambridge Face Memory Test Inverted Version
DDTFS: Disjunctive dual-target face search

## Footnotes

1 These data are not the focus of the present study. However, analysis of the rating taken across Experiments 1 and 2 confirms the participants reported the three conditions as being different. Formal analysis shows a significant effect of the Group (*F*(2,200) = 8.69, *p* < .001, ${\eta }_{G}^{2}$ = .08). Faces used as targets for the Typical group were rated as more similar (*M* = 2.43, *SE* = 0.16) than those used in the Distinctive (*M* = 1.91, *SE* = 0.13, *p* = .018) or Mixed groups (*M* = 1.67, *SE* = 0.09, *p* < .001). There was no significant difference in the similarity of target faces used in the Distinctive and Mixed groups (*p* = .56).

2 Prioritisation was determined by performance across all trials, however breaking the data down into 4 epochs shows that prioritisation remained constant across the duration of the study.

3 As might be expected, 23/32 participants in the Mixed group prioritised the distinctive over the typical face target. (The normal approximation to the binomial distribution shows the probability of prioritising the distinctive face over the typical face was *p* = .01. )The comparison was not conducted for the Distinctive or Typical group as the difference in ratings for faces within each of these groups was marginal.

4 As might be expected, in the Mixed group. 23/34 participants prioritised the distinctive over the typical face target. (The normal approximation to the binomial distribution shows the probability of prioritising the distinctive face over the typical face was (*p* = 0.02).) An independent t test comparing proportion accuracy for Non-prioritised distinctive (*M* = 0.91, *SE* = 0.02) and typical (*M* = 0.87, *SE* = 0.03) faces did not reach significance (*t*(33) = 1.37, *p* = 0.18, *d* = 0.24).

## References

[CR1] Anwyl-Irvine, A., Dalmaijer, E. S., Hodges, N., & Evershed, J. (2020). Realistic precision and accuracy of online experiment platforms, web browsers, and devices. *Behavior Research Methods,**53*, 1407–1425. 10.3758/s13428-020-01501-5 33140376 10.3758/s13428-020-01501-5 PMC8367876

[CR2] Bäckman, L. (1991). Recognition memory across the adult life span: The role of prior knowledge. *Memory & Cognition,**19*, 63–71. 2017030 10.3758/BF03198496

[CR3] Bakeman, R. (2005). Recommended effect size statistics for repeated measures designs. *Behavior Research Methods,**37*, 379–384. 10.3758/BF03192707 16405133 10.3758/BF03192707

[CR4] Bindemann, M., Sandford, A., Gillatt, K., Avetisyan, M., & Megreya, A. M. (2012). Recognising Faces seen alone or with others: Why are two heads worse than one? *Perception,**41*(4), 415–435. 10.1068/p6922 22896915 10.1068/p6922

[CR5] Bowles, D. C., McKone, E., Dawel, A., Duchaine, B., Palermo, R., Schmalzl, L., Rivolta, C., Wilson, C. E., & Yovel, G. (2009). Diagnosing prosopagnosia: Effects of ageing, sex, and participant-stimulus ethnic match on the Cambridge Face Memory Test and Cambridge Face Perception Test. *Cognitive Neuropsychology,**26*(5), 423–455. 19921582 10.1080/02643290903343149

[CR6] Bruce, V., Henderson, Z., Greenwood, K., Hancock, P. J. B., Burton, A. M., & Miller, P. (1999). Verification of face identities from images captured on video. *Journal of Experimental Psychology: Applied,**5*(4), 339–360. 10.1037/1076-898X.5.4.339 10.1037/1076-898X.5.4.339

[CR7] Burton, A. M., Jenkins, R., Hancock, P. J., & White, D. (2005). Robust representations for face recognition: The power of averages. *Cognitive Psychology,**51*(3), 256–284. 10.1016/j.cogpsych.2005.06.003 16198327 10.1016/j.cogpsych.2005.06.003

[CR8] Burton, A. M., White, D., & McNeill, A. (2010). The Glasgow face matching test. *Behavior Research Methods,**42*(1), 286–291. 10.3758/BRM.42.1.286 20160307 10.3758/BRM.42.1.286

[CR9] Cohen, M. E., & Carr, W. J. (1975). Facial recognition and the von Restorff effect. *Bulletin of the Psychonomic Society,**6*(4), 383–384. 10.3758/BF03333209 10.3758/BF03333209

[CR10] Cowan, N. (1988). Evolving conceptions of memory storage, selective attention, and their mutual constraints within the human information-processing system. *Psychological Bulletin,**104*(2), 163–191. 10.1037/0033-2909.104.2.163 3054993 10.1037/0033-2909.104.2.163

[CR11] Cowan, N. (1998). *Attention and memory: An integrated framework*. Oxford University Press.

[CR12] Davis, J. P., Forrest, C., Treml, F., & Jansari, A. (2018). Identification from CCTV: Assessing police super-recogniser ability to spot faces in a crowd and susceptibility to change blindness. *Applied Cognitive Psychology,**32*(3), 337–353. 10.1002/acp.3405 10.1002/acp.3405

[CR13] Davis, J. P., & Valentine, T. (2009). CCTV on trial: Matching video images with the defendant in the dock. *Applied Cognitive Psychology,**23*(4), 482–505. 10.1002/acp.1490 10.1002/acp.1490

[CR14] Duchaine, B., & Nakayama, K. (2006). The Cambridge Face Memory Test: Results for neurologically intact individuals and an investigation of its validity using inverted face stimuli and prosopagnosic participants. *Neuropsychologia,**44*(4), 576–585. 10.1016/j.neuropsychologia.2005.07.001 16169565 10.1016/j.neuropsychologia.2005.07.001

[CR15] Dunn, J. D., Kemp, R. I., & White, D. (2021). Top-down influences on working memory representations of faces: Evidence from dual-target visual search. *Quarterly Journal of Experimental Psychology,**74*(8), 1368–1377. 10.1177/17470218211014357 10.1177/17470218211014357 33899599

[CR16] Ellis, H. D., Shepherd, J. W., & Davies, G. M. (1979). Identification of familiar and unfamiliar faces from internal and external features: Some implications for theories of face recognition. *Perception,**8*(4), 431–439. 10.1068/p08043 503774 10.1068/p08043

[CR17] Ellis, H. D., Shepherd, J. W., Gibling, F., & Shepherd, J. (1988). Stimulus factors in face learning. In *Practical aspects of memory: Current research and issues, Vol. 1: Memory in everyday life* (pp. 136–144). Wiley.

[CR18] Faul, F., Erdfelder, E., Lang, A. G., & Buchner, A. (2007). G^*^Power 3: A flexible statistical power analysis program for the social, behavioral, and biomedical sciences. *Behavior Research Methods,**39*, 175–191. 17695343 10.3758/BF03193146

[CR19] Kazak, A. E. (2018). Editorial: Journal article reporting standards. *American Psychologist,**73*(1), 1–2. 10.1037/amp0000263 29345483 10.1037/amp0000263

[CR20] Kelley, M. R., & Nairne, J. S. (2001). von Restorff revisited: Isolation, generation, and memory for order. *Journal of Experimental Psychology: Learning, Memory, and Cognition,**27*, 54–66. 10.1037/0278-7393.27.1.54 11204107 10.1037/0278-7393.27.1.54

[CR21] Kemp, R., Towell, N., & Pike, G. (1997). When Seeing should not be believing: Photographs, credit cards and fraud. *Applied Cognitive Psychology,**11*(3), 211–222. 10.1002/(SICI)1099-0720(199706)11:3%3c211::AID-ACP430%3e3.0.CO;2-O 10.1002/(SICI)1099-0720(199706)11:3<211::AID-ACP430>3.0.CO;2-O

[CR22] Lewin, C., & Herlitz, A. (2002). Sex differences in face recognition- women’s faces make the difference. *Brain and Cognition,**50*(1), 121–128. 10.1016/S0278-2626(02)00016-7 12372357 10.1016/S0278-2626(02)00016-7

[CR23] Light, L. L., Kayra-Stuart, F., & Hollander, S. (1979). Recognition memory for typical and unusual faces. *Journal of Experimental Psychology: Human Learning and Memory,**5*(3), 212–228. 10.1037/0278-7393.5.3.212 528913 10.1037/0278-7393.5.3.212

[CR24] Lobmaier, J. S., & Mast, F. W. (2007). Perception of novel faces: The parts have it! *Perception,**36*(11), 1660–1673. 10.1068/p5642 18265846 10.1068/p5642

[CR25] Megreya, A. M., & Burton, A. M. (2006). Unfamiliar faces are not faces: Evidence from a matching task. *Memory & Cognition,**34*(4), 865–876. 10.3758/BF03193433 17063917 10.3758/BF03193433

[CR26] Megreya, A. M., Bindemann, M., & Havard, C. (2011). Sex differences in unfamiliar face identification: Evidence from matching tasks. *Acta Psychologica,**137*(1), 83–89. 10.1016/j.actpsy.2011.03.003 21459354 10.1016/j.actpsy.2011.03.003

[CR27] Meissner, C. A., & Brigham, J. C. (2001). Thirty years of investigating the own-race bias in memory for faces: A meta-analytic review. *Psychology, Public Policy, & Law,**7*, 3–35. 10.1037/1076-8971.7.1.3

[CR28] Mestry, N., Menneer, T., Cave, K. R., Godwin, H. J., & Donnelly, N. (2017). Dual-target cost in visual search for multiple unfamiliar faces. *Journal of Experimental Psychology: Human Perception and Performance,**43*(8), 1504–1519. 10.1037/xhp0000388 28368160 10.1037/xhp0000388

[CR29] Moore, K. N., & Lampinen, J. M. (2019). The role of attention and memory in search for missing persons. *Journal of Applied Research in Memory and Cognition,**8*(2), 189–201. 10.1016/j.jarmac.2019.01.005 10.1016/j.jarmac.2019.01.005

[CR30] Newell, F. N., Chiroro, P., & Valentine, T. (1999). Recognizing unfamiliar faces: The effects of distinctiveness and view. *The Quarterly Journal of Experimental Psychology Section A,**52*(2), 509–534. 10.1080/713755813 10.1080/713755813 10428688

[CR31] Pavlovia. (2020) [software]. https://pavlovia.org

[CR32] Peirce, J. W. (2007). PsychoPy - Psychophysics software in Python. *Journal of Neuroscience Methods,**162*(1–2), 8–13. 10.1016/j.jneumeth.2006.11.017 17254636 10.1016/j.jneumeth.2006.11.017 PMC2018741

[CR33] Qualtrics XM Platform. (2020). [software]. https://qualtrics.com

[CR34] R Core Team. (2021). R: A language and environment for statistical computing. R Foundation for Statistical Computing, Vienna, Austria. http://www.R-project.org/

[CR35] Russell, R., Duchaine, B., & Nakayama, K. (2009). Super-recognizers: People with extraordinary face recognition ability. *Psychonomic Bulletin & Review,**16*, 252–257. 10.3758/PBR.16.2.252 19293090 10.3758/PBR.16.2.252 PMC3904192

[CR36] Schmidt, S. R. (1991). Can we have a distinctive theory of memory? *Memory & Cognition,**19*, 523–542. 10.3758/BF03197149 1758300 10.3758/BF03197149

[CR37] Smillie, E., Mestry, N., & Donnelly, N. (2022). *The impact of target distinctiveness and conceptual information on the discrimination of unfamiliar faces.* Unpublished manuscript.

[CR38] Sporer, S. L. (2001). Recognizing faces of other ethnic groups: An integration of theories. *Psychology, Public Policy, & Law,**7*, 36–97. 10.1037/1076-8971.7.1.36

[CR39] Thielgen, M. M., Schade, S., & Bosé, C. (2021). Face processing in police service: The relationship between laboratory-based assessment of face processing abilities and performance in a real-world identity matching task. *Cognitive Research: Principles and Implications,**6*(1), 54. 10.1186/s41235-021-00317-x 34351527 10.1186/s41235-021-00317-x PMC8342700

[CR40] Towler, J., Kelly, M., & Eimer, M. (2016). The focus of spatial attention determines the number and precision of face representations in working memory. *Cerebral Cortex,**26*(6), 2530–2540. 10.1093/cercor/bhv083 25903465 10.1093/cercor/bhv083

[CR41] Valentine, T. (1991). A unified account of the effects of distinctiveness, inversion, and race in face recognition. *The Quarterly Journal of Experimental Psychology Section A,**43*(2), 161–204. 10.1080/14640749108400966 10.1080/14640749108400966 1866456

[CR42] White, D., Kemp, R. I., Jenkins, R., Matheson, M., & Burton, A. M. (2014). Passport officers’ errors in face matching. *PLoS ONE,**9*(8), e103510. 10.1371/journal.pone.0103510 25133682 10.1371/journal.pone.0103510 PMC4136722

[CR43] Wickham, H. (2016). *ggplot2: Elegant graphics for data analysis*. Springer.

[CR44] Wolfe, J. M. (2012). Saved by a log: How do humans perform hybrid visual and memory search? *Psychological Science,**23*(7), 698–703. 10.1177/0956797612443968 22623508 10.1177/0956797612443968 PMC3966104

[CR45] Wolfe, J. M. (2021). Guided Search 6.0: An updated model of visual search. *Psychonomic Bulletin and Review,**28*, 1060–1092. 10.3758/s13423-020-01859-9 33547630 10.3758/s13423-020-01859-9 PMC8965574

[CR46] Wright, D. B., Boyd, C. E., & Tredoux, C. G. (2003). Inter-racial contact and the own-race bias for face recognition in South Africa and England. *Applied Cognitive Psychology,**17*, 365–373. 10.1002/acp.898

[CR47] Wright, D., Boyd, C. E., & Tredoux, C. (2001). A field study of own-race bias in South Africa and England. *Psychology Public Policy and Law,**7*(1), 119–133. 10.1037/1076-8971.7.1.119 10.1037/1076-8971.7.1.119

[CR48] Yin, R. K. (1969). Looking at upside-down faces. *Journal of Experimental Psychology,**81*(1), 141. 10.1037/h0027474 10.1037/h0027474

[CR49] Zoom Video Communications, Inc. (2020). *ZOOM cloud meetings* (Version 4.6.9) [Mobile app]. App Store. https://apps.apple.com/us/app/zoom-cloud-meetings/id546505307

